# The Complete Mitochondrial Genome of *Pennella* sp. Parasitizing *Thunnus albacares*

**DOI:** 10.3389/fcimb.2022.945152

**Published:** 2022-06-30

**Authors:** Hongyan Liu, Zhengyi Fu, Shengjie Zhou, Jing Hu, Rui Yang, Gang Yu, Zhenhua Ma

**Affiliations:** ^1^ Tropical Aquaculture Research and Development Center, South China Sea Fisheries Research Institute, Chinese Academy of Fishery Sciences, Sanya, China; ^2^ Sanya Tropical Fisheries Research Institute, Sanya, China; ^3^ Key Laboratory of South China Sea Fishery Resources Exploitation and Utilization, Ministry of Agriculture and Rural Affairs, Guangzhou, China

**Keywords:** mitochondrial genome, *Thunnus albacares*, parasite, *Pennella*, copepod.

## Abstract

In the study, the parasite from the yellowfin tuna (*Thunnus albacares*) was separated, and morphological observation and molecular identification were carried out. Our results showed that the parasite was similar to *Pennella* sp. Its cephalothorax was covered by spherical to spherical non-branched nipples of almost the same size, which were very similar in shape and arrangement. A pair of slightly larger, the unbranched antenna was present on the outer margin of the small papillae-covered area. The gene sequence of COX1 with a length of 1,558 bp in the mitochondria of the parasite was 100% similar to *Pennella* sp. (MZ934363). The mitochondrial genome had a total length of 14,620 bp. It consisted of 36 genes (12 protein-coding, 22 transfer RNAs and 2 ribosomal RNAs) and a dummy control region, but the mitochondrial genome had no ATP8 gene. Morphological observation showed that *Pennella* sp. was dark red, with a convex cephalothorax, with a total length of 8.42 cm, parasitic on the dorsal side of yellowfin tuna. *Pennella* sp. included the cephalothorax, neck, trunk, abdomen and egg belt. This study was the first report on the mitochondrial genome of *Pennella* sp. The results provide basic data for further identifying the parasites of *Pennella* genus.

## Introduction

*Pennella* is a parasite belonging to Copepod that usually lives in large pelagic fishes (Hogans et al., 1985; Suyama et al., 2020). After metamorphosis, the female enters the host’s body surface to parasitize while the male swims freely (Hogans et al., 1985). Their bodies were straight and slender, with feathery bellies and straight egg sacs (Hogans, 2017). People usually judge the species of *Pennella* from the host, the size and number of antennal processes, the shape and arrangement of cephalothorax, etc. Due to the wide variety of species, there was no specific standard to determine the identity (Suyama et al., 2021). Suyama et al. conducted morphological and genetic analysis on 52 *Pennella* individuals from 12 final hosts, of which 29 were identified as large species, 20 as small and medium-sized species and three as small new species. The 17 of them were identified as *P. balaenoptera*, *P. filosa*, *P. instructa*, and *P. benzi*. Nine samples were identified as *P. filosa*. Three samples could not be determined as *P. balaenoptera*. The remaining 23 were unidentified species (Suyama et al., 2021). The systematic classification of these parasites is limited, and the complete genome of the parasite hasn’t been reported. Mitochondrial genome is a unique and easily accessible genetic marker of organisms (Brown et al., 1979). The complete mitochondrial genome can be used to distinguish parasites, phylogeny (Mueller et al., 2004; Lei et al., 2017), and population genetic structure, it is important to characterize the genome of the Copepod.

The hosts of *Pennella* are very diverse, including a variety of teleost fishes and even marine mammals (Suyama et al., 2021). The host yellowfin tuna (*Thunnus albacares*) in this study belongs to the mackerel family and tuna genus. Yellowfin tuna, a member of the scombroid family of tuna, has a distinct north-south migration habit, and its torpedo-like body shape enables it to move quickly to catch food. Yellowfin tuna were found in the tropical and subtropical waters of the Pacific, Indian and Atlantic oceans, and in the South China Sea and near Taiwan (Sund et al., 1981). It is a higher economic value nourishing treasure due to its high flesh quality and rich nutrition, deeply loved by people. Food safety is also the focus of people’s attention. We need to detect the species of parasites it infects to expand the understanding of fish vulnerable to parasites. Although tuna can be bred in captivity, the low survival of fish during artificial breeding continually hinders the tuna aquaculture industry. Up to the present, 80% of cultured fish were from wild juveniles, and wild fish can carry pathogens, viruses and parasites, which is not conducive to the smooth progress of subsequent breeding. Therefore, it is urgent to supplement the data on the parasites of yellowfin tuna.

In our study, we carried out morphological observation and molecular identification to determine its species. The results of this study provide a fundamental basis for identifying parasites in yellowfin tuna and other fish, expand people’s understanding of parasites, and lay a foundation for the occurrence and prevention of parasites. The complete mitochondrial genome sequence of *Pennella* sp. provides primary data for understanding the genomic diversity and evolution of fish copepods as well as for studying new genetic markers for population genetics and species identification.

## Materials and Methods

### Source of Materials

**Figure 1:**
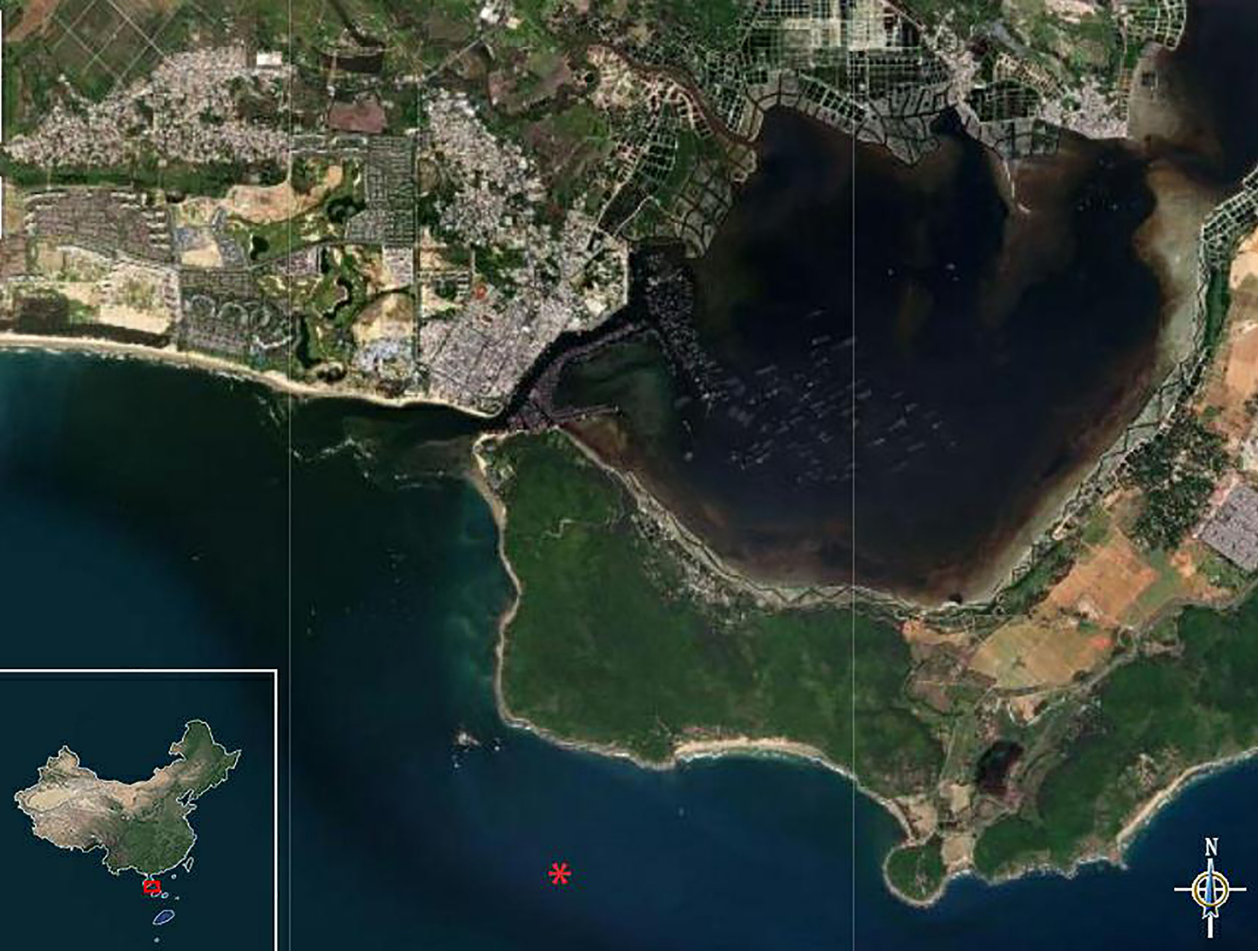
Sampling point bitmap, the sampling point is “*” in the figure.

In our study, the parasite-attached yellowfin tuna was caught near Xincun Harbour, Hainan, China (E108°80’17’’, N18°50’15’’). **Figure 1** showed the offshore area where this yellowfin tuna was caught. It was artificially fished and transported to the Tropical Aquaculture Research and Development Center, South China Sea Fisheries Research Institute, Chinese Academy of Fishery Sciences. The parasite-infected yellowfin tuna was found to be on the verge of death. The parasite was found in the muscles near the dorsal fin of the dying yellowfin tuna. We carefully collected it on the surface of the fish with tweezers and carefully observed it in a clean petri dish.

### Morphological Observation and Whole Mitochondrial Genome Sequencing of *Pennella* sp.

We first measured the length of the parasite from the front end of the head to the back end of the abdomen and observed whether the abdomen had egg belts and raised tentacles on the cephalothorax. Then, we placed it under the anatomical microscope (Olympus SZ40, Japan), observed its morphological characteristics, pressed the cephalothorax, trunk and abdomen, observed and photographed. After observation, the whole parasite was quickly frozen with liquid nitrogen and stored at -80°C for standby. In order to further understand the genetic status and evolution of mitochondria, we studied and obtained the complete mitochondrial genome of *Pennella* sp. Genomic DNA of the parasite was extracted from muscle using Marine Animal Tissue Genomic DNA Extraction Kit (Tiangen Biochemical Technology Co., Ltd, China). DNA was processed by Shanghai Lingen Biotechnology Co., Ltd. and it was paired-end sequenced by Illumina NovaSeq 6000 sequencing technology, using SPAdes v3. 14.1 the software splices clean data. For most species, ring maps are usually used to display the basic research results of the genome in the first genome research. According to the assembled genome sequence of the sequenced sample, combined with the prediction results of the coding gene, the sample genome is displayed in a circle (**Figure 3**). The software used is CGView. Finally, the results were compared with the sequences reported in NCBI GenBank database(http://blast.ncbi.nlm.nih.gov/Blast.cgi), and the phylogenetic tree was constructed using Mega 5.2 software (Hall, 2005).

## Results

### Parasite Attachment Site

**Figure 2:**
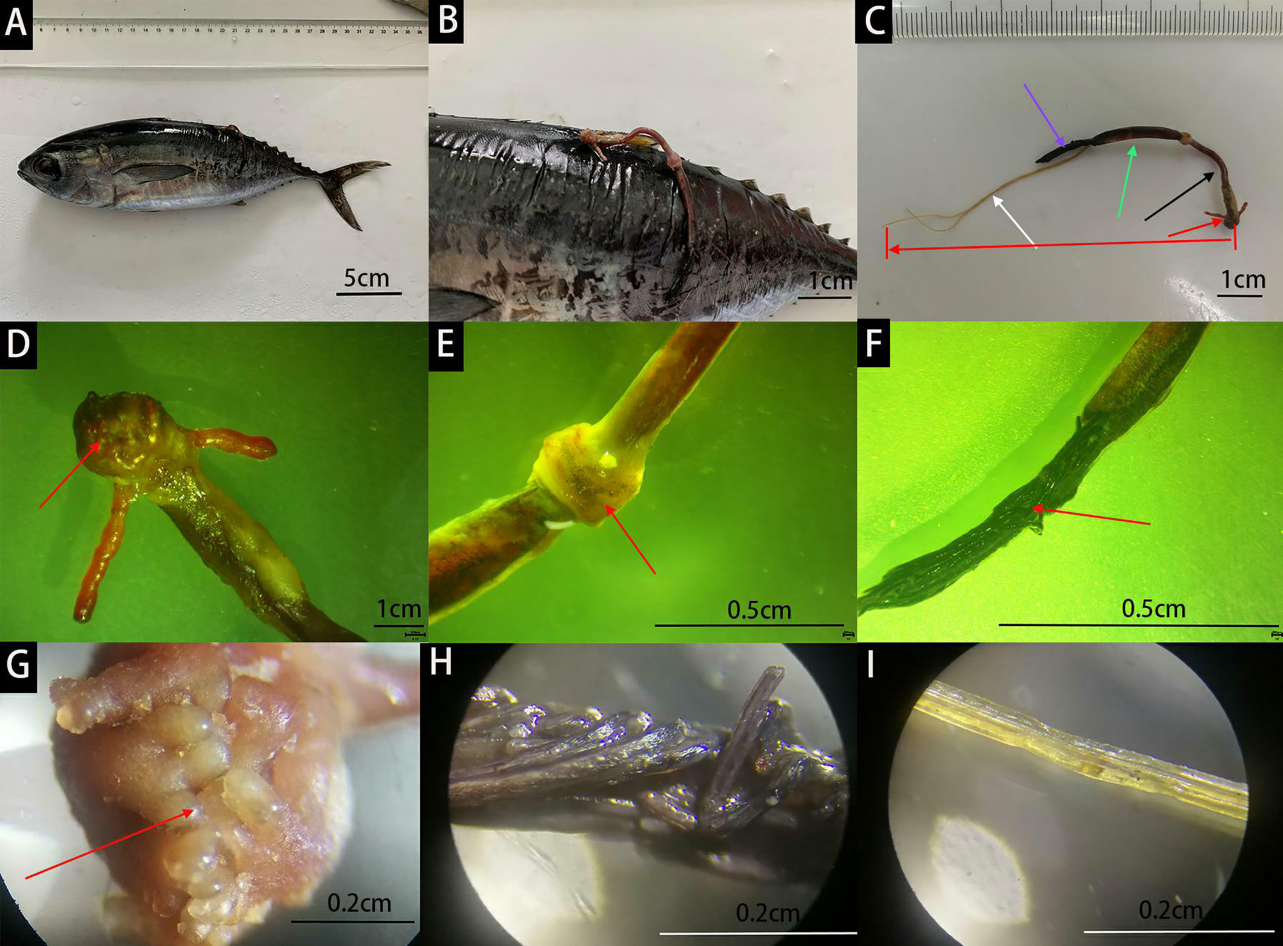
Infected yellowfin tuna **(A)**. Parasites on the posterior dorsal fin **(B)**. The red arrow represents the cephalothorax, the black arrow represents the neck, the green arrow represents the trunk, the purple arrow represents the abdomen, and the white arrow represents the egg belts **(C)**. The head and tail of the red arrow at the bottom of the figure represent the starting and ending positions of the parasite **(C)**. Mastoid process on the cephalothorax **(D, G)**. The node between the neck and trunk **(E)** Abdomen **(F, H)**. Yellow egg belts **(I)**.

According to the observation of dead yellowfin tuna, it was found that the parasite is mainly attached to 1/2 of the base to the end of the dorsal fin of yellowfin tuna, on the back half of yellowfin tuna (**Figures 2A, B**). When the parasite is attached to the fish body, it entered the fish body from the chest to the body, while other parts were exposed to the water and could swing with the water flow.

### Parasite Appearance Description

The parasite in this study can be divided into five parts: cephalothorax, neck, trunk, abdomen and egg belt (**Figure 2C**). The cephalothorax was covered by several equally distributed mastoids of similar size. The heads of the mastoid were light yellow, and the roots were red (**Figure 2G**). There was a pair of large antennae without branches below (**Figure 2D**). The larger antennae were located at the lateral edge of the cephalothorax. Below the cephalothorax was the neck and trunk, between which there was a node (**Figure 2E**), below the trunk was the abdomen (**Figure 2F**), which was composed of several black strips (**Figure 2H**). Below the abdomen were two yellow egg belts (**Figure 2I**). The body length of the parasite in this study was 5.25 cm, egg band 4.49 cm, cephalothorax 0.39 cm, neck 1.79 cm, body 1.88 cm, and abdomen 1.21 cm.

### Complete Mitogenome and Molecular Identification

**Figure 3:**
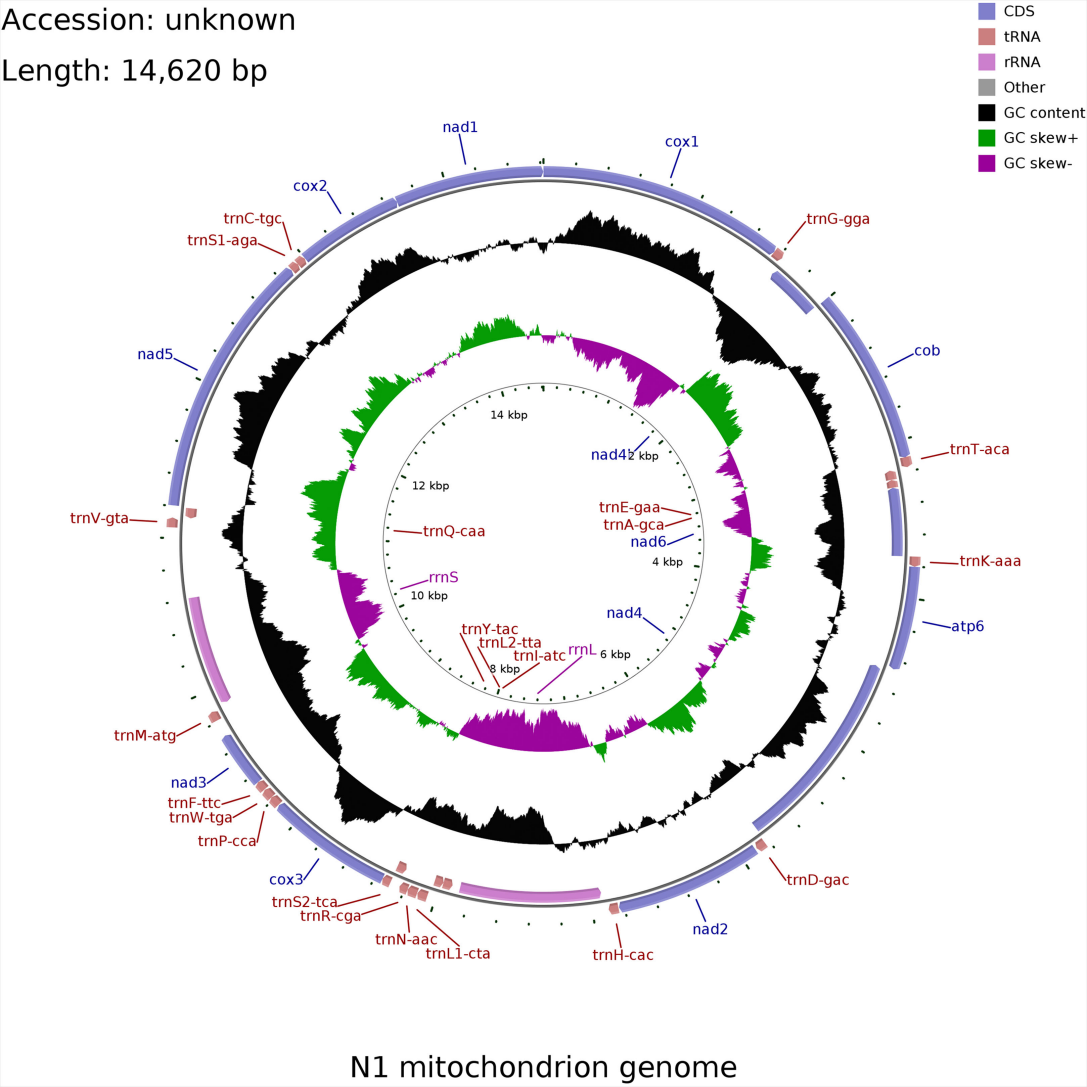
Gene map of the *Pennella* sp. complete mitochondrial genome.

The of the parasite was 14,620 bp in length. According to the genome circle map (**Figure 3**), the structure of the genome was including two rRNA genes, 12 protein-coding genes (lack of *ATP8*), 22 tRNA genes, two rRNA genes, a light-strand replication origin (OL), and a putative control region (CR). The overall base composition was 31.9% of A, 31.9% of T, 18% of C, and 18% of G with a slight C+G bias (36.0%) like other vertebrate mitochondrial genomes.

For the 12 protein-coding genes, 5 genes began with TTG and 5 began with ATT, while only *COB* and *NAD3* began with ATG and ATA, respectively. Eight genes shared a stop codon TAA (*NAD4L*, *COB*, *COX2*, *NAD1*, *NAD2*, *NAD3*, *NAD4* and *NAD5*), three have TAG (*NAD6*, *ATP6*, *COX3*), and *COX1* has an incomplete stop codon. It had two non-coding regions, the L-strand replication origin region (350 bp) located between *COX1* and *COB*, and the control region (449 bp) located within the *COB* and *ATP6*.

**Figure 4:**
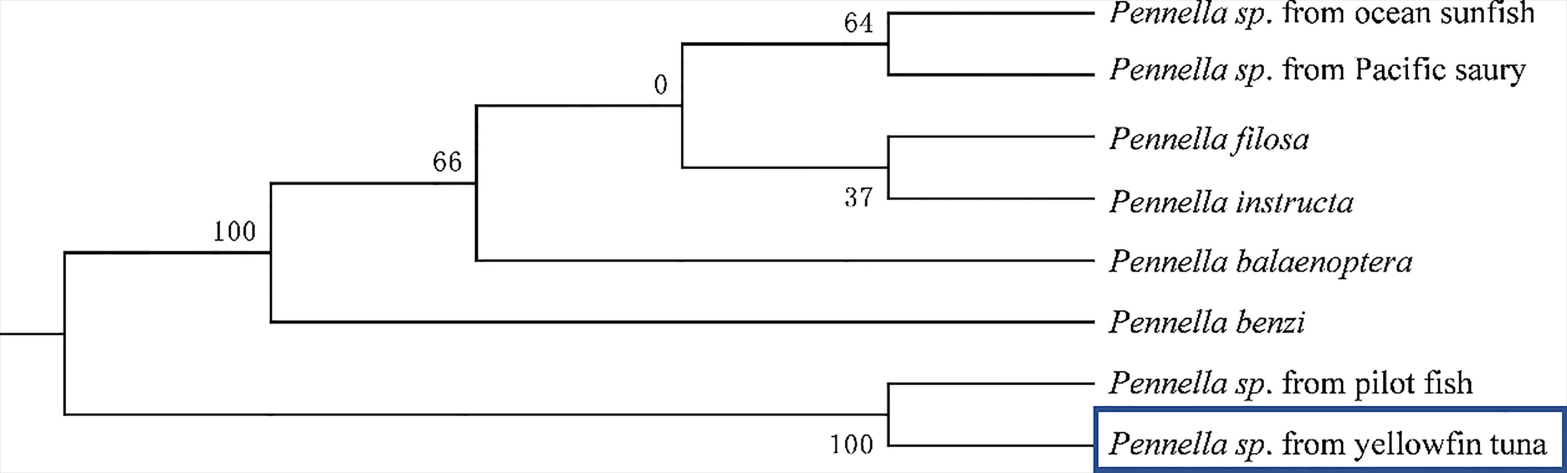
The phylogenetic relationship was estimated using the Maximum Likelihood method for the *COX1* genes. Genbank accession numbers: *Pennella* sp. from pilot fish (MZ934363), *Pennella benzi* (LC642589), *Pennella* sp. from Pacific saury (LC638600), *Pennella* sp. from ocean sunfish (LC638579), *Pennella filosa* (LC642600), *Pennella instructa* (LC642628), *Pennella balaenoptera* (MG701292).

After gene sequencing, we compared the *COX1* gene (1,558 bp) with NCBI database,

and found that the parasite was closely related to *Pennella* sp., with a similarity of 100%. We reconstructed the phylogenetic relationship between the species and other species. The phylogenetic tree was constructed by the adjacency method (**Figure 4**). The species of parasite samples could not be determined.

## Discussion

Current studies have shown that copepods of the genus *Pennella* were parasites of marine aquatic organisms (e.g., cephalopods, pelagic fish, whales). Most *Pennella* were parasitic on marine fish (Suyama et al., 2021). They can infect fish of economic interest, including tuna and swordfish. They penetrate the muscles of the host, seriously damage the internal organs, and grow by absorbing the nutrients in the fish, resulting in fish stress response, loss of appetite, changes in swimming speed, and threatening important functions such as heart, intestine and stomach (Mugetti et al., 2021). We can see from the previous summary that *Pennella* sp. from North Pacific armorhead lacks oocysts due to incomplete development; *Pennella* sp. from Pacific sury and *Pennella* sp. from Japanese amberjack contain two or three pairs of large antennal processes with branches on the outer edge of the cephalothorax (Suyama et al., 2021). However, the parasite has only a pair of non-branching antennal processes and egg belts, which were not consistent with the above characteristics. It is concluded that this parasite was different from the species previously studied. By comparing this parasite with other species, it was found that the parasites in this study have similarities with *Pennella* sp., a parasite parasitic on pilot fish (Suyama et al., 2021). The COX1 gene of the parasite in this study was compared with the genome of *Pennella* sp., and the similarity of gene COX1 sequence with *Pennella* sp. found by predecessors was 100%, but the species was unknown. At present, there are few reports on the large parasites on the body surface of yellowfin tuna.

After gene annotation, it was found that *Pennella* sp. in this study had no ATP8 gene. In copepods, the complete mitochondrial genome of *Eurytemora affinis*, *Tigriopus kingsejongenesis* and *Calanus sinicus* contains the gene ATP8. The whole mitochondrial genome length of the above copepods was 14,900-16,700 bp, which is longer than that of the parasite (Wang et al., 2011; Choi et al., 2019; Hwang et al., 2019). Studies have reported that this gene is not found in *Diphyllobothrium latum* (Park et al., 2007), parasitic flatworm (Tang et al., 2017; Zhang et al., 2017), *Benedenia humboldti* n. sp. (Baeza et al., 2019), Cestode (Kim et al., 2007). The whole mitochondrial genome lengths of the above flatworms, tapeworms and nematodes were 13,400-14,660 bp. It can be concluded that the length of species with ATP8 gene in mitochondrial genome was longer than that without ATP8 gene. The lack of ATP8 gene was a unified feature of flatworms. It may be that the evolution process needs to adapt to the environment, or the gene ATP8 degenerates in some species (Barat et al., 2012). The specific reasons need to be further explored and studied. Whether it is also a characteristic of *Pennella* is still unknown and needs to be further studied.

## Conclusions

Through morphological observation, *Pennella* sp. in this study was composed of cephalothorax, neck, trunk, abdomen and egg belt. The mitochondrial genome had a total length of 14,620 bp, including 2 rRNA genes, 22 tRNA genes and 12 protein coding genes (lack of ATP8 gene), a light-strand replication origin (OL), and a putative control region (CR). The COX1 gene with a length of 1558bp in the whole genome had a high similarity with *Pennella* sp. (MZ934363), which was 100%. This study provides basic data for the further development of yellowfin tuna and fills the academic gap.

## Data Availability Statement

The data presented in the study are deposited in the GenBank, accession number ON161759.

## Author Contributions

GY and RY: conceptualization. HL and ZF: experimental operation. HL and JH: field sampling. HL and ZF: sample determination. HL: writing – original draft preparation. ZM and SZ: writing – review and editing. All authors read and approved the final manuscript.

## Funding

This work was supported by Hainan Major Science and Technology Project (ZDKJ2021011); Central Public-interest Scientific Institution Basal Research Fund, CAFS (2020TD55) and Central Public-Interest Scientific Institution Basal Research Fund South China Sea Fisheries Research Institute, CAFS (2021SD09).

## Conflict of Interest

The authors declare that the research was conducted in the absence of any commercial or financial relationships that could be construed as a potential conflict of interest.

## Publisher’s Note

All claims expressed in this article are solely those of the authors and do not necessarily represent those of their affiliated organizations, or those of the publisher, the editors and the reviewers. Any product that may be evaluated in this article, or claim that may be made by its manufacturer, is not guaranteed or endorsed by the publisher.
